# Impact of Surface Coatings on the Photocatalytic Activity and Cytotoxicity of Titanium Dioxide Nanoparticles in Human Keratinocytes: Implications for Sunscreen Safety

**DOI:** 10.1021/acsomega.5c13018

**Published:** 2026-06-05

**Authors:** Maria Eleni Katsanou, Philipp-Kjell Ficht, Lienhard Wegewitz, Jörg Adams, Lars Böckmann, Steffen Emmert, Wolfgang Maus-Friedrichs

**Affiliations:** † Clausthaler Center for Materials Technology, 26534 Clausthal University of Technology, Clausthal-Zellerfeld 38678, Germany; ‡ Clinic and Policlinic for Dermatology, Venereology and Allergology, University Medical Center, Rostock 18057, Germany; § Institute of Physical Chemistry, Clausthal University of Technology, Clausthal-Zellerfeld 38678, Germany

## Abstract

Titanium dioxide nanoparticles (TiO_2_ NPs) are widely used in sunscreens as UV filters because of their high UV absorption efficiency. However, their potential to generate reactive oxygen species (ROS) under UV irradiation raises concerns about their photocatalytic activity and associated cytotoxicity. This study investigates how surface coatings, specifically SiO_2_ and Al_2_O_3_–SiO_2_, affect the photocatalytic performance and biological impact of anatase-dominant TiO_2_ NPs on human HaCaT keratinocytes. Photocatalytic activity was evaluated via methylene blue degradation under UVA (365 nm) and UVB (290 nm) irradiation, showing that uncoated TiO_2_ NPs (Aeroxide P25) exhibited significantly higher degradation rates (64%) compared to SiO_2_-coated (59–55%) and Al_2_O_3_–SiO_2_-coated (24–22%) variants. Cytotoxicity assays (XTT) revealed that UVA exposure alone had a minimal effect, but in combination with anatase-rich TiO_2_ NPs, a dose- and time-dependent reduction in cell viability was observed. Notably, Al_2_O_3_–SiO_2_-coated NPs showed significantly lower cytotoxicity than SiO_2_-coated or uncoated anatase-based NPs. ROS measurements confirmed elevated oxidative stress in nanoparticle-exposed cells under UVA, although no significant differences were observed between coating types. Raman spectroscopy revealed structural damage to cellular components in cells exposed to both UVA and TiO_2_ NPs, suggesting membrane disruption and genotoxic potential. These results underscore the importance of coating chemistry in nanomaterial design and call for more realistic testing paradigms involving formulated products and complex skin models to inform regulatory standards and consumer safety.

## Graphical abstract

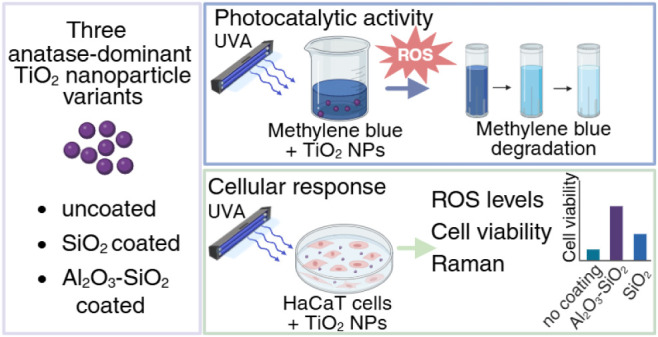

## 1 Introduction

The objective of the present study is 2-fold. First, it seeks to assess the impact of coatings on the photocatalytic activity of anatase-based titanium dioxide nanoparticles used as UV filters in commercial sunscreens. Second, it aims to determine the toxic potential of coated and uncoated mixtures of anatase-rich titanium dioxide nanoparticles toward human cells, given their potential health risks.

Sunscreens are widely used skin care products to reduce the risk for skin cancer and photoaging. The combination of ultraviolet (UV) radiation to which people are exposed to with the increasing prevalence of dermatological diseases on a yearly basis^1^ leads to an increased necessity for the use of sunscreens. Sunscreens comprise two categories of UV filters: “organic” filters and inorganic UV filters.^2^ Among the inorganic materials, zinc oxide and titanium dioxide are commonly used.^3,4^ Titanium dioxide in the form of nanoparticles is a highly sought-after ingredient for sunscreens due to its ability to provide strong UV protection mainly through UV absorption.^5^ The electron system of nano-TiO_2_ can be readily excited by UV light, resulting in the generation of reactive oxygen species (ROS).

These ROS then interact with cellular macromolecules, including proteins and nucleic acids, which can potentially result in DNA damage and other adverse effects.^6^ Moreover, the presence of titanium in nanosize makes it transparent, which is a cosmetic benefit when compared with traditional ingredients that are less esthetically pleasing.^7^ However, nanoparticles have the capacity to penetrate the skin via three primary pathways: (1) the transappendageal route, which refers to penetration through sweat pores, hair follicles, and sebaceous glands; (2) the transcellular route, where penetration directly through skin cells is possible; and (3) the intercellular route, where they navigate between skin cells.^7,8^

Once inside the skin, the nanoparticles may encounter living cells and potentially interact with them. It is important to note that the potential toxicity of nanoparticles on human health has been studied,^9^ and, more specifically, TiO_2_ NPs have been found to have mild toxicity and the capacity to cause oxidative DNA damage due to ROS production.^10^

In a review article, Cao et al.^11^ examined the existing in vivo and in vitro studies concerning the genotoxicity of TiO_2_ NPs. The authors emphasized the interaction of these particles with DNA and the induction of reactive oxygen species (ROS), which, when considered collectively, have the capacity to compromise DNA repair mechanisms and induce chromosomal damage.^11^ In a similar way, Vaudagna et al. reported considerable toxicity when Aeroxide P25 was applied to both prokaryotic and eukaryotic (blood) cells.^12^ Currently, nano-TiO_2_ is extensively used as a UV filter in sunscreens due to its ability to minimize the whitening effect on skin.^13^

Nanoparticles are increasingly coated to prevent possible cell damage. The typical coating of these nanoparticles with materials such as SiO_2_, Al_2_O_3_, or a combination thereof is designed to enhance dispersion and reduce photocatalytic activity.^14–17^ For instance, surface modification with methacryloxypropyltrimethoxysilane (MCPTMS) has been shown to reduce TiO_2_ photocatalytic activity by 72% without compromising transparency, as demonstrated by Siddiquey et al.^18^ According to the findings of Jacobs et al.,^19^ such coatings have been shown to enhance the stability of nanoparticle formulations and to reduce their photoactivity. However, it should be noted that significant heterogeneity can result from variations in coating material, thickness, and embedding matrix.^19^ This variability complicates risk assessment and regulatory efforts and contributes to the growing number of “known unknowns” in the field of nanomaterial safety.^20^

Concerns regarding the safety of TiO_2_ NPs in cosmetic applications are a matter of wide recognition within the scientific community, with a consensus that further research is urgently required.^10,11^ Baek et al. investigated the impact of surface coatings on the antibacterial activity of TiO_2_ nanoparticles against *E. coli*, finding that silica-coated TiO_2_ NPs generated significantly higher levels of reactive oxygen species (ROS), indicating enhanced surface reactivity.^21^ Previous studies have shown that the photocatalytic and biological behavior of TiO_2_ nanoparticles is strongly influenced by crystal phase and surface treatment. Parkin et al.^22^ examined a broad and heterogeneous set of inorganic particles contained in sunscreens but were chemically distinct from those investigated in this work, while Damiani et al.^23^ investigated commercial TiO_2_ dispersions in dermal fibroblasts and both suggested that only rutile-phase TiO_2_ nanoparticles should be used in sunscreens. In contrast, the present study focuses on a more defined comparison between uncoated Evonik Aeroxide TiO_2_ P25 and mixed-phase anatase/rutile TiO_2_ nanoparticles coated with SiO_2_ or Al_2_O_3_–SiO_2_. We further used HaCaT keratinocytes as an epidermal cell model more directly relevant to initial topical exposure and applied a lower nanoparticle concentration (200 μg/mL) to investigate coating-dependent effects under more conservative in vitro conditions. Neither Parkin et al. nor Damiani et al. included Raman spectroscopy, and its incorporation here provides an additional molecular-level dimension to the evaluation of coating-dependent effects under UVA exposure.

Although SCCS guidance has favored predominantly rutile, low-photocatalytic TiO_2_ crystal phase for cosmetic use,^24^ XRD-based analyses of commercial sunscreen products indicate that crystal-phase composition is not always uniform and that anatase-rich TiO_2_ nanoparticles are still detected in marketed formulations.^25,26^ Investigation of anatase-containing systems therefore remains scientifically relevant, particularly for understanding how surface coatings modulate photocatalytic activity and the associated biological responses. The present study integrates the comparison of photocatalytic activity, cytotoxicity, intracellular ROS generation, and Raman-detectable cellular biochemical alterations within the same experimental framework.

## 2 Materials and Methods

### 2.1 Titanium Dioxide Nanoparticles

Three types of anatase/rutile titanium dioxide nanoparticles were purchased for the experimental study (see Table 1).

**Table 1:** Summary of Anatase/Rutile Titanium Dioxide Nanoparticle Types Used in This Study

Crystal phase	Ratio	Coating	Average particle size (nm)	Purchased from
Anatase/Rutile (Aeroxide P25)	80%/20%	no	21	Sigma-Aldrich
Anatase/Rutile	80%/20%	Al_2_O_3_–SiO_2_	21	Nanostructured & Amorphous Materials, Inc.
Anatase/Rutile	90%/10%	SiO_2_	21	Nanostructured & Amorphous Materials, Inc.

### 2.2 Physical Characterization of Titanium Dioxide Particles

X-ray diffraction (XRD) analysis was performed using an Empyrean diffractometer (Malvern Panalytical) equipped with a Cu Kα radiation source. The crystal phase composition of the samples was determined from the diffraction patterns, and quantitative phase analysis of the anatase/rutile mixtures was carried out using the Spurr and Myers equation.^27^ Particle size information was based on the manufacturers’ specifications.

### 2.3 Determination of Photocatalytic Activity by Methylene Blue Tests and UV–vis Spectroscopy

The above-mentioned TiO_2_ nanoparticles (NPs) were incorporated into water-based suspensions. Equal volumes of each nanoparticle suspension were mixed with a methylene blue (MB) solution to ensure consistency across samples. Titanium dioxide is a well-known photocatalyst; upon exposure to ultraviolet (UV) light, it generates reactive oxygen species (ROS), which have the capacity to degrade organic compounds such as methylene blue. This degradation process was employed as a model reaction to assess and compare the photocatalytic activity of the different nano-TiO_2_ formulations.^28,29^ Although an exact match in anatase/rutile ratio between the mixed-phase samples would have been desirable, all investigated mixtures were anatase-dominant (≥80% anatase). Previous work has suggested that in this composition range the rutile fraction is too low to substantially promote electron–hole separation, and the photocatalytic behavior is therefore largely governed by the anatase phase. Accordingly, modest differences in rutile content within this anatase-rich range are not expected to fundamentally alter the overall photocatalytic response.^30^

Each experiment was conducted over a 3 h period, during which UV–vis absorption spectra were recorded every 15 or 30 min for in total of 3 h, using a Jasco UV-670 UV–vis spectrophotometer. Experiments were performed in triplicate for each formulation, both in the presence and absence of light. The solution for each measurement consisted of 1 g of the TiO_2_ suspension (0.035 g/L) mixed with 1 g of a methylene blue solution (10 ppm). The solution was stirred continuously for 3 h. The photocatalytic activity of the TiO_2_ nanoparticles was assessed by monitoring methylene blue (MB) degradation under UV irradiation. Two irradiation conditions were applied: UVA (365 nm) and short-wavelength UVB (290 nm). These wavelengths were selected to compare wavelength-dependent differences in photocatalytic activity under controlled laboratory conditions. The 365 nm condition served as a defined UVA exposure, with a measured irradiance of 2.56 mW/cm^2^, and was chosen as a relevant UVA wavelength because UVA represents the major fraction of solar UV radiation reaching the Earth’s surface.^28^ By contrast, the 290 nm condition, with a measured irradiance of 1.05 mW/cm^2^, was included as a shorter-wavelength, higher-energy UVB exposure for comparative purposes only. Degradation rates (DR) of methylene blue were calculated using the following equation:^29^

$$Degradation\;Rate\;\%=\left(\frac{A_{0}-A}{A_{0}}\right)\times 100$$

where *A*_0_ is the initial absorbance and *A* is the absorbance of methylene blue after the reaction. The absorbance values used for the calculations represent the mean of each sample prior to normalization.

### 2.4 Preparation of Cell Cultures

Human keratinocyte cell line (HaCaT) was cultivated in Dulbecco’s Modified Eagle Medium (DMEM) supplemented with 10% fetal bovine serum (FBS) and 1% penicillin/streptomycin. HaCaT cells were passaged twice per week after reaching 70–80% confluence. Cells were washed three times with phosphate-buffered saline (PBS) before enzymatically detached with trypsin/EDTA for 6 min at 37 °C in a 5% CO_2_ atmosphere and diluted in fresh medium afterward before seeding in a new cell culture flask. For experiments, aliquots of 50 μL cell suspension were mixed with 5 mL of CASYTon to determine cell count using the CASY cell count system (OLS). Cell suspensions were then diluted to the desired concentration for seeding in a well plate format. For the 6-well format, the cell suspension was diluted to a final concentration of 1 × 10^5^ cell/mL and seeded at 2 × 10^5^ cells per well. After 24 h cells adhered to the well surface and the old medium was removed. Fresh medium containing 200 μg/mL TiO_2_–NPs was added to incubate for another 24 h.

Cells were washed three times with PBS before UV exposure using a Bio-Link UV-Cross-linker (Vilber, Germany) equipped with 5 UV lamps for UVA (T-8.L, 365 nm, 8 W power each). Cells were exposed to 10 J/cm^2^ of UVA at a 15 cm distance from the light sources. The radiation doses were chosen to resemble 30–60 min (UVA) direct sun exposure on an average summer day in Vienna.^31^ After another 24 h, cells were enzymatically detached as described above. Samples were centrifuged for 5 min at 250 × *g* and RT before resuspending the cell pellets in fresh medium again for transport.

### 2.5 Cytotoxicity Analysis

HaCaT cells were seeded similarly as described above in 96-well plates with 10^4^ cells per well in 100 μL. TiO_2_–NP stocks were ultrasonicated for 20–30 min before dilution in cell culture medium to a concentration of 200 μg/mL and added to the cells at time points varying from 0.5 to 24 h before washing the excess TiO_2_–NPs with PBS three times. Then HaCaT cells were exposed to different doses of UVA (0, 10, 15 J/cm^2^) and left to incubate for 48 h. Afterward, the XTT assay was performed according to the manufacturer’s instructions by mixing 6 parts of XTT labeling reagent and 1 part electron coupling reagent, before adding 70 μL of the XTT labeling mix to each well. After 4 h of incubation in the dark at 37 °C and 5% P­(CO_2_) absorbance at 450 nm and 650 nm as reference was quantified.

### 2.6 Quantification of Intracellular Reactive Oxygen Species (ROS) by H_2_DCFDA Assay

For intracellular ROS quantification, HaCaT cells were seeded into black 96-well plates with transparent bottoms, using the same protocol as for cytotoxicity testing. TiO_2_–NPs solutions were prepared as described above at a final concentration of 200 μg/mL and incubated with the cells for 24 h. The fluorescent probe H_2_DCFDA was dissolved in DMSO to obtain a 2.5 mM stock solution and diluted in PBS to a working concentration of 25 μM. The culture medium was removed, and the cells were washed once with PBS. Then, 100 μL of the H_2_DCFDA working solution was added per well. Plates were incubated for 45 min in the dark at 37 °C and 5% CO_2_ to allow uptake and intracellular conversion by cellular esterases. Subsequently, the solution was removed, the cells were washed again with PBS, and fresh medium was added. The cells were then exposed to UVA radiation (0, 2.5, or 5 J/cm^2^), and positive controls were treated with 0.035% H_2_O_2_. Fluorescence intensity was measured 2 h after treatment at 488 nm excitation and 535 nm emission.

### 2.7 Statistical Analysis

Raw data were corrected for background noise by subtracting the absorbance of the culture medium mixed with the XTT labeling mix. Afterward, metabolic activity was calculated relative to the untreated control that was not exposed to UV radiation. During each experimental run, all treatments were conducted as triplicates that were used to calculate a mean for the experiment. The means of three independent experiments (*n* = 3) were tested for normality by Shapiro–Wilk test. After confirmation of data normality, one-way ANOVA followed by Dunnett’s test (post hoc) was used to find statistically significant differences to the untreated control that was exposed to the same UVA dose.

### 2.8 Raman Spectroscopy Measurements

Raman spectra were recorded using a Vertex 70v spectrometer (Bruker Optics GmbH & Co., Ettlingen, Germany) coupled to a Senterra microscope and equipped with a CCD detector (1024 × 256 pixels). Excitation was provided by a 532 nm laser operated at 20 mW, and measurements were performed using 20x and 50x bright-field objectives. The instrument was calibrated with calcium carbonate prior to analysis. Spectral processing was performed using OPUS 7.6 software. Background subtraction was applied to all spectra, followed by normalization to the Raman band at 1354 cm^–1^. This band, assigned to DNA- and protein-related contributions, was selected because it was consistently present and of high intensity in all spectra.

## 3 Results and Discussion

### 3.1 Comparison of Photocatalytic Activity of Al_2_O_3_/SiO_2_- and SiO_2_-Coated Nano-TiO_2_ with Uncoated TiO_2_ Nanoparticles

#### 3.1.1 Degradation Experiments with Methylene Blue

Titanium dioxide (TiO_2_) nanoparticles have been demonstrated to absorb ultraviolet (UV) light, which, in turn, has been shown to induce the formation of reactive oxygen species (ROS). Because of their high reactivity, ROS are effective at degrading a wide range of organic compounds. Utilizing this property, the degradation of methylene blue dye was employed as a model reaction to evaluate the photocatalytic activity of the various nanoparticles described above, with particular attention to the influence of surface coatings. The experiment was conducted over a period of 3 h, during which UV–vis spectra were recorded at 15 or 30 min intervals. Each condition was tested in triplicate, in both the presence and absence of light. Two UV light sources were utilized: 365 nm (UVA) and 290 nm (UVB).

##### 3.1.1.1 UVA Light Source (365 nm)

The results are presented in Figure 1, in which the change in absorption at 664 nm was measured, corresponding to the characteristic absorption peak of methylene blue (MB). The diagram encompasses the degradation of MB in the presence of uncoated and coated anatase-rich mixture of anatase/rutile nano-TiO_2_. It is notable that include control spectra collected in the absence of UV light. This control step is critical to confirm that any observed changes in the degree of absorption of light are solely due to photocatalytic activity under UVA (365 nm) irradiation. In the absence of light, the UV–vis spectra remained stable, showing only a minimal decrease in absorbance (∼5%) over the 3 h period. This indicates negligible photolytic decomposition or thermal degradation of methylene blue (MB), confirming that significant degradation occurs only under ultraviolet (UV) irradiation.^32^ It is observed that under UVA exposure, there are clear differences in photocatalytic activity. For uncoated nanoparticles, methylene blue degradation was substantial: a 64% reduction in concentration was measured after 3 h in the presence of Aeroxide P25 (a mixed-phase TiO_2_ composed of anatase and rutile). It has been reported that anatase generally exhibits higher photocatalytic activity than rutile.^33,34^

**Figure 1:**
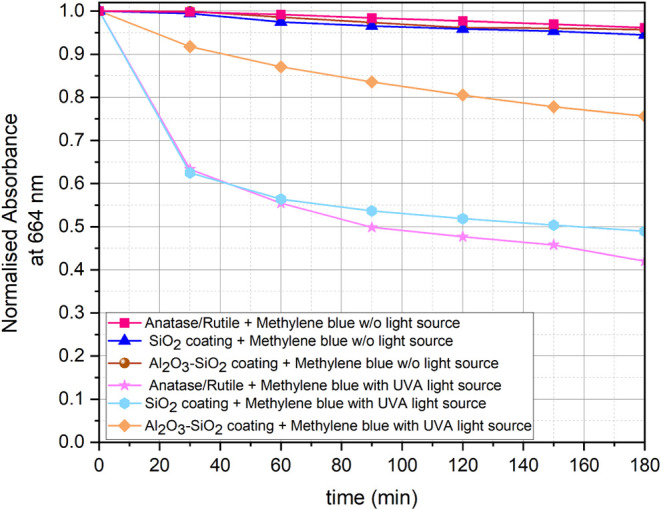
Absorbance at 664 nm as a proxy for photocatalytic activity of coated and uncoated mixture of anatase/rutile-based TiO_2_ nanoparticles, in the presence and absence of UVA irradiation.

However, some studies have shown that Aeroxide P25 displays enhanced photoreactivity due to a synergistic effect between its anatase and rutile phases.^35–37^ In our case, this effect is not observed, likely because, according to Su et al., the anatase-to-rutile ratio for efficient methylene blue photo-oxidation is approximately 60:40.^30^

In contrast, the presence of surface coatings reduced the photocatalytic performance of the TiO_2_ nanoparticles. With the SiO_2_ coating, the concentration of methylene blue (MB) decreased by 59% during the same period, while the application of a double coating of Al_2_O_3_–SiO_2_ significantly reduced degradation, with a decrease in MB concentration of only 24% after 3 h. The lower degradation rate observed for the Al_2_O_3_–SiO_2_ coating compared with SiO_2_ alone suggests reduced ROS production when aluminum oxide is present. This agreed with the findings of Parkin et al.,^22^ who also observed that aluminum oxide-coated rutile nanoparticles exhibited lower photocatalytic activity. Results of another study indicate that SiO_2_ coatings may be less effective in reducing ROS production due to the possible formation of Ti–O–Si chemical bonds.^21^

##### 3.1.1.2 UVB Light Source (290 nm)

The same set of measurements was repeated by using a UVB light source (290 nm). For this particular wavelength, UV–vis spectra were recorded at 15 min intervals over a total duration of 3 h. Absorbance at 664 nm was monitored as this corresponds to the characteristic absorption peak of methylene blue (MB). Figure 2 presents MB degradation in the presence of uncoated and coated anatase-based nano-TiO_2_. As for the UVA experiments, in the absence of light, the UV–vis spectra remained stable, with only a minor decrease in absorption (around 5% over a period of 3 h), indicating negligible photolytic or thermal degradation of MB. This finding indicates that degradation occurs exclusively under UV irradiation.^32^

**Figure 2:**
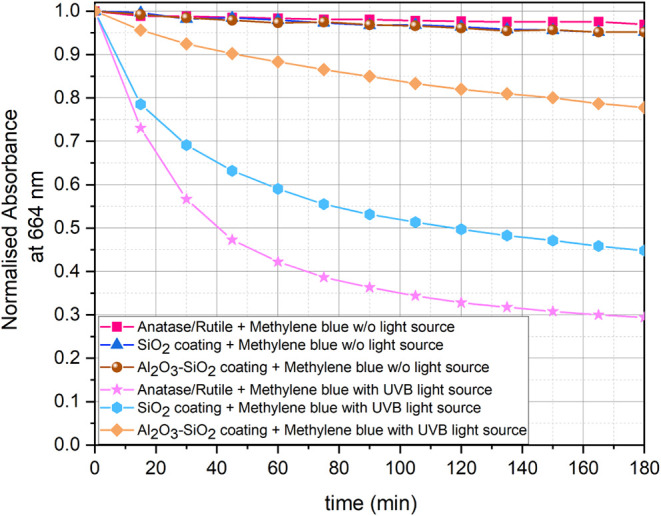
Absorbance at 664 nm as a proxy for photocatalytic activity of coated and uncoated mixture of anatase/rutile-based TiO_2_ nanoparticles, in the presence and absence of UVA irradiation.

It was evident that there were marked variations in photocatalytic activity when the samples were exposed to UVB light. For uncoated nanoparticles, a substantial degradation of methylene blue was recorded, with a 70% reduction in MB concentration after 3 h for Aeroxide P25 (a mixed-phase TiO_2_). Conversely, surface coatings significantly diminished the photocatalytic efficiencies of the samples. When coated with SiO_2_, the MB concentration decreased by 55% over the same period. The dual Al_2_O_3_–SiO_2_ coating exhibited a more pronounced inhibitory effect, with only a 22% reduction in MB concentration being observed after three h.

The findings of this study indicate that surface modifications can significantly alter the photocatalytic behavior of nano-TiO_2_, likely by limiting the generation or transport of reactive oxygen species (ROS) at the particle–solution interface.

A comparison of the two types of UV radiation revealed that UVB light was more effective than UVA in promoting the degradation of MB, particularly for uncoated anatase-based nanoparticles. MB degradation increased by 6% in the Aeroxide P25 sample under UVB compared with that under UVA. It is interesting to note that the differences for anatase-rich-coated nanoparticles are as follows: with the SiO_2_–Al_2_O_3_ coating, MB degradation increased by only 2% under UVB, while for the SiO_2_ coating, a 4% increase was observed. The results obtained emphasize the role of surface coatings in modulating photocatalytic performance under different irradiation conditions.

### 3.2 Particle Coating and Time-Dependent Toxicity (XTT Assay)

In vitro experiments were conducted using HaCaT cell cultures, selected specifically due to their relevance as immortalized human keratinocytes. HaCaT cells are widely used as a model for studying epidermal homeostasis,^38^ skin barrier function,^39^ and various dermatological pathologies.^40^ In this study, cell cultures treated with 3 different types of anatase-based TiO_2_ nanoparticles, both coated and uncoated, were exposed to different intensities of UVA. Following exposure, cellular metabolic activity was assessed by the XTT assay to evaluate the impact of UV radiation in the presence of nanoparticles. The results were compared to determine which nanoparticle type, coating, and UV dose had the most significant effect on cell viability.

The results are presented in Figure 3. In these experiments, HaCaT cells were incubated with either SiO_2_- or Al_2_O_3_–SiO_2_-coated anatase-based TiO_2_ nanoparticles (NPs) at a concentration of 200 μg/mL for 30 min, 3, 6, 12, 18, or 24 h. After washing with PBS twice, cells were irradiated with UVA (0, 10, or 15 J/cm^2^) in reduced medium volume, followed by replenishment with fresh medium and a 48-h incubation. Metabolic activity was quantified using the XTT assay and normalized to untreated control cells (no TiO_2_ NPs, no UVA).

**Figure 3:**
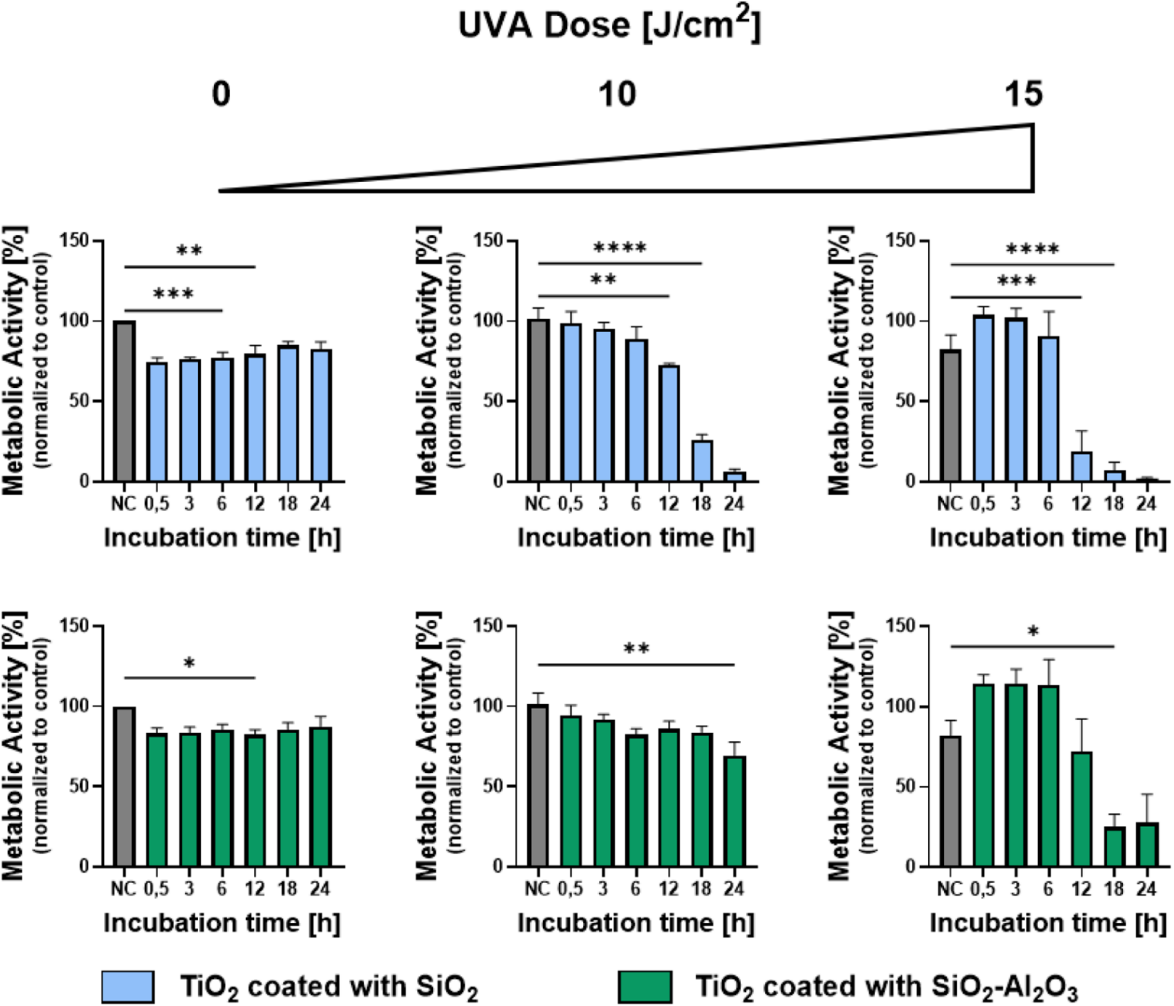
Influence of coated anatase-rich TiO_2_–NP incubation time and irradiation dose on UVA-induced cytotoxicity in HaCaT cells. Data are presented as mean ± SD (*n* = 3). Statistical significance was determined by one-way ANOVA followed by Dunnett’s post hoc test (n.s., not significant; **p < 0.01; ***p < 0.001; ****p < 0.0001). NC: negative control.

UVA irradiation without the addition of anatase-rich TiO_2_–NPs led to no significant reduction in cell viability with 82% metabolic activity remaining even after 15 J/cm^2^ UVA exposure. Anatase-based TiO_2_ NPs without UVA light (0 J/cm^2^) caused a slight time-dependent reduction in viability. For SiO_2_-coated anatase-rich TiO_2_ NPs, viability was 75% after 30 min of incubation but gradually increased to 83% at 24 h. Similarly, SiO_2_–Al_2_O_3_-coated anatase-based TiO_2_ NPs reduced viability to 83% at 30 min, which recovered to 87% at 24 h. In both cases, viability remained above the DIN ISO 10993-5 cytotoxicity threshold (70%), indicating no cytotoxicity in the absence of UVA, although statistically significant differences were observed to untreated controls.

When 10 J/cm^2^ of UVA irradiation was applied, cytotoxic effects emerged for anatase-based TiO_2_-treated cells. For SiO_2_-coated anatase-rich TiO_2_ NPs, the viability decreased significantly to 73% at 12 h, 26% at 18 h, and 6% at 24 h. For SiO_2_–Al_2_O_3_-coated anatase-based TiO_2_ NPs, viability declined more slowly, reaching 69.5% at 24 h. Both coated particles significantly reduced viability compared with UVA controls without TiO_2_ NPs. Notably, the SiO_2_ coating caused significant cytotoxicity after 12 h, whereas the SiO_2_–Al_2_O_3_ coating remained only slightly cytotoxic after 24 h based on the DIN ISO 10993-5 70% criterion. At 15 J/cm^2^ of UVA, cytotoxicity was more pronounced. For SiO_2_-coated anatase-based TiO_2_ NPs, viability dropped to 18% at 12 h, 7% at 18 h, and 2% at 24 h. For SiO_2_–Al_2_O_3_-coated anatase-based TiO_2_ NPs, viability decreased to 25% at 18 h and 28% at 24 h. These results are similar to those achieved with 10 J/cm^2^ but with a higher degree of cell death. Higher UVA doses were consistently associated with an increased cytotoxicity. While the SiO_2_–Al_2_O_3_ coating reduced cell damage compared to SiO_2_-coated nanoparticles, it did not prevent it.

In summary, UVA radiation alone did not cause a significant cytotoxicity. Both SiO_2_- and SiO_2_–Al_2_O_3_-coated anatase-based TiO_2_ NPs caused moderate, noncytotoxic viability loss without UVA. However, in combination with UVA, anatase-based TiO_2_ NPs became strongly cytotoxic with an increasing UVA dose and increasing incubation time. The SiO_2_–Al_2_O_3_ coating significantly reduced but did not prevent the cytotoxicity caused by UVA irradiation, in full agreement with the results of the methylene blue degradation experiments.

#### 3.2.1 Cytotoxicity Comparison between Coated and Uncoated Particles (XTT Assay)

Toxicity of uncoated and coated anatase-based TiO_2_–NPs was compared at equal incubation times and UVA doses to find potential differences. The results are presented in Figure 4. In these experiments, cells were incubated with either Aeroxide P25 or SiO_2_ or SiO_2_–Al_2_O_3_-coated anatase/rutile mixture of TiO_2_ NPs (200 μg/mL) for 30 min, 12 h, or 24 h. After washing with PBS, cells were irradiated with UVA (0, 10 J/cm^2^) in reduced medium volume, followed by medium replenishment and a 48-h incubation. Metabolic activity was assessed using the XTT assay and normalized to untreated control cells (no TiO_2_–NPs, no UVA).

**Figure 4:**
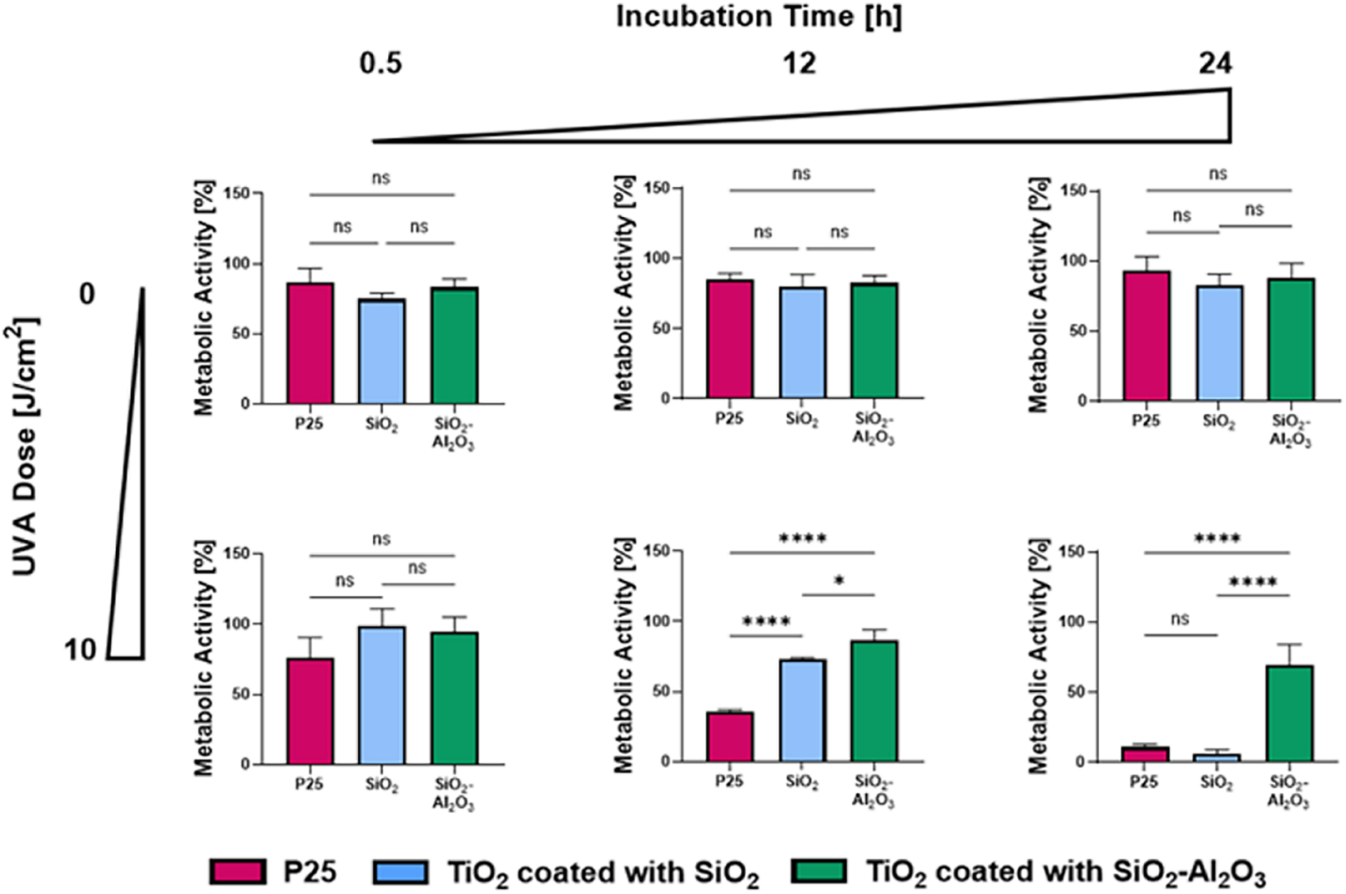
Comparison of UVA-induced cytotoxicity of uncoated and coated anatase-based TiO_2_ NPs in HaCaT cells. Data are shown as mean ± SD (*n* = 3). Statistical significance was assessed using one-way ANOVA followed by Tukey’s multiple comparisons post hoc test. n.s., not significant; * *p* < 0.05; **p < 0.01; ***p < 0.001; ****p < 0.0001.

UVA radiation is essential for TiO_2_–NP-induced cytotoxicity. In the absence of UVA, all three tested anatase-based TiO_2_ nanoparticle (NP) types; Aeroxide P25, SiO_2_-coated, and SiO_2_–Al_2_O_3_-coated, exert minimal effects on cell viability. Short incubation times, even with UVA exposure, do not result in significant toxicity. After 30 min of incubation under UVA (10 J/cm^2^), viability values for coated particles were 99% (SiO_2_-coated) and 95% (SiO_2_–Al_2_O_3_-coated), exceeding those for uncoated particles, Aeroxide P25 (76%), but all remained above the ISO 10993-5 cytotoxicity threshold of 70%. These results indicate that both photoactivation and a sufficient exposure duration are required to induce measurable cytotoxic effects.

After prolonged incubation (12 h) with UVA, pronounced differences emerged between coated and uncoated NP mixtures. Uncoated TiO_2_ anatase-rich nanoparticles showed the greatest toxicity, with the viability reduced to 36% for Aeroxide P25. Surface coatings substantially mitigated these effects, with SiO_2_-coated particles showing 73% viability and SiO_2_–Al_2_O_3_-coated particles maintaining 86% viability. At 24 h of UVA exposure, all NP types became cytotoxic, but the SiO_2_–Al_2_O_3_ coating retained the most protective capacity, maintaining viability close to 69.5%, whereas Aeroxide P25 and SiO_2_-coated anatase-based NPs all dropped below 12%. Despite this, all values at 24 h fell below the ISO 70% viability threshold.

#### 3.2.2 Levels of Reactive Oxygen Species within Cells

For measuring the levels of reactive oxygen species (ROS) within cells the H_2_DCFDA assay, also known as the DCFDA assay, was used. The results are presented in Figure 5. For these experiments, cells were incubated with either Aeroxide P25 or SiO_2_- or SiO_2_–Al_2_O_3_-coated anatase-based TiO_2_–NPs (200 μg/mL) for 12 h. After washing with PBS, cells were irradiated with UVA (0, 2.5, or 5 J/cm^2^) in reduced medium volume, followed by medium replenishment and a 3-h incubation. ROS production was assessed using the H_2_DCFDA assay and data from untreated control cells (without H_2_DCFA) were subtracted from mean fluorescence intensities as background.

**Figure 5:**
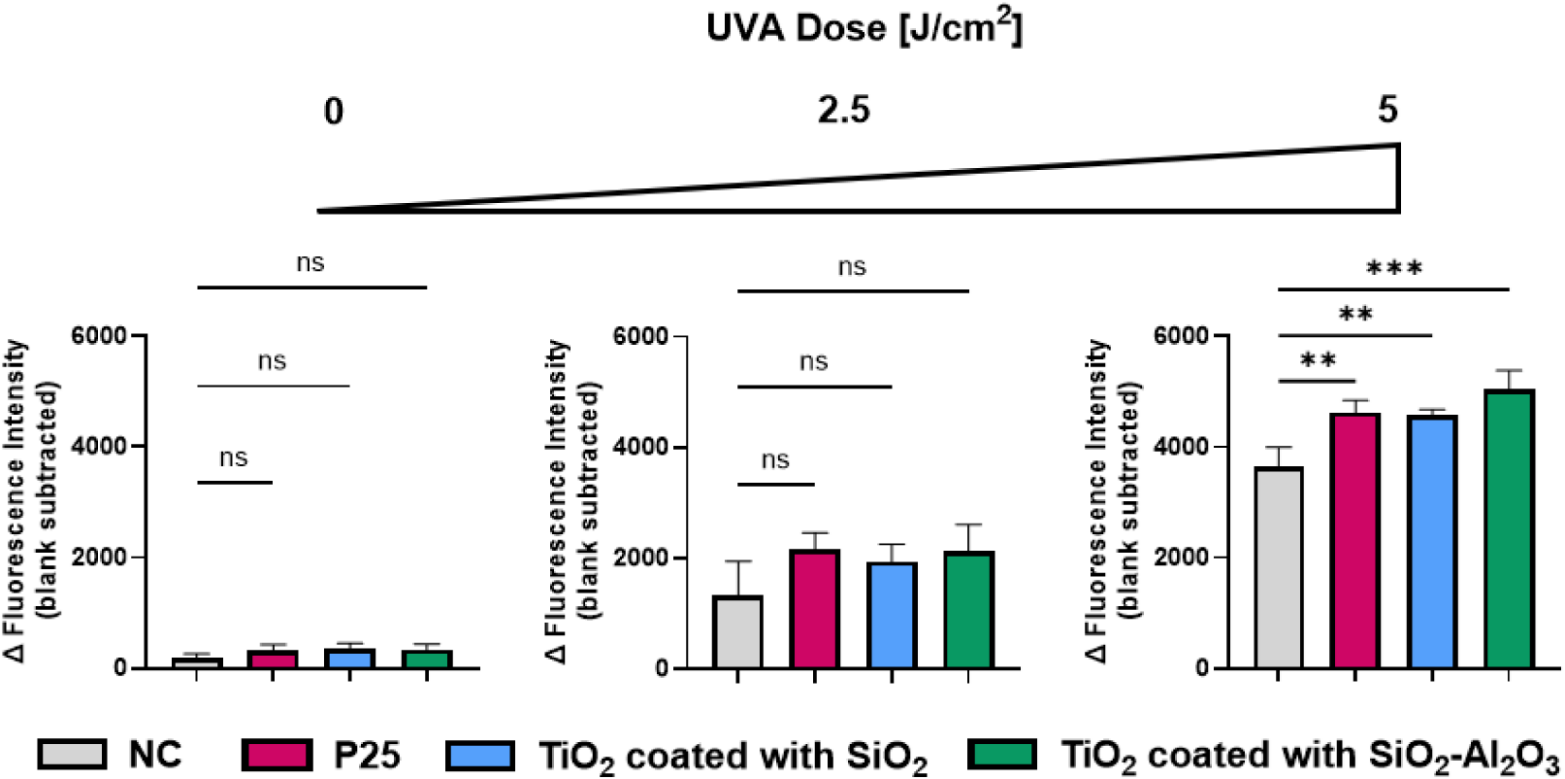
UVA-induced ROS production by TiO_2_ NPs in HaCaT cells. Data are shown as mean ± SD (*n* = 3). Statistical significance was assessed using one-way ANOVA followed by Dunnett’s post hoc test. n.s., not significant; ***p < 0.001.

Without UVA exposure, ROS production did not differ between control cells and those treated with anatase-based TiO_2_ NPs. At 2.5 J/cm^2^ UVA, no statistically significant changes were observed, although a slight trend toward increased H_2_DCFDA fluorescence intensity was noted for all three anatase-based TiO_2_ NP types. At 5 J/cm^2^ UVA, ROS levels increased significantly for all NP-treated groups compared to controls, rising from baseline values (∼3560 RFU) to ∼5000 RFU at maximum. No significant differences in ROS generation were detected between the different anatase-based TiO_2_ nanoparticle formulations.

This indicates that photocatalytic ROS production was induced by UVA irradiation but did not vary between NP types, possibly because of the relatively low UVA doses used or limitations in the sensitivity of the H_2_DCFDA assay.

In summary, UVA exposure resulted in a clear, dose-dependent increase in ROS, from 171 RFU (no UVA) to 1317 RFU (2.5 J/cm^2^) and 3560 RFU (5 J/cm^2^). Addition of anatase-rich TiO_2_–NPs caused significant increases after 5 J/cm^2^ UVA; however, regardless of the anatase-based TiO_2_ NP surface coating, this effect can be attributed to challenges in detection and the presence of a high background signal induced by UVA exposure.

### 3.3 Raman Measurements of Cell Cultures Treated with Coated and Uncoated Nano-TiO_2_

In this study, human keratinocyte (HaCaT) cells were incubated in PBS solution stored in centrifuge tubes, with screw cap and subsequently analyzed with Raman spectroscopy to evaluate the effects of UVA radiation, anatase-based TiO_2_ nanoparticles (NPs), and their combined exposure. The cell cultures were treated with 10 J/cm^2^ UVA irradiation and with 200 μg of anatase-based TiO_2_ nanoparticles. Following the observed decrease in cell viability, we aimed to identify the underlying mechanisms—specifically, whether the treatments primarily impact cellular lipids, proteins, DNA, or a combination thereof.

Figure 6 presents the normalized Raman spectra of HaCaT cells subjected to four treatment conditions: untreated ones, UVA exposure alone, exposure to anatase-based TiO_2_ nanoparticles, and combined exposure to anatase-based TiO_2_ nanoparticles and UVA. The primary objective of these experiments is to evaluate cellular interactions with nanoparticles under UVA irradiation, thereby mimicking exposure scenarios such as those encountered with sunscreen use if the nanoparticles manage to diffuse through the outer skin layer to living cells. Given that titanium dioxide nanoparticles are widely used as UV filters in cosmetic formulations and that their combination with UVA was observed to reduce cell viability, these analyses aim to elucidate the specific biomolecular components such as DNA, proteins, or lipids that are affected by these interactions.

**Figure 6:**
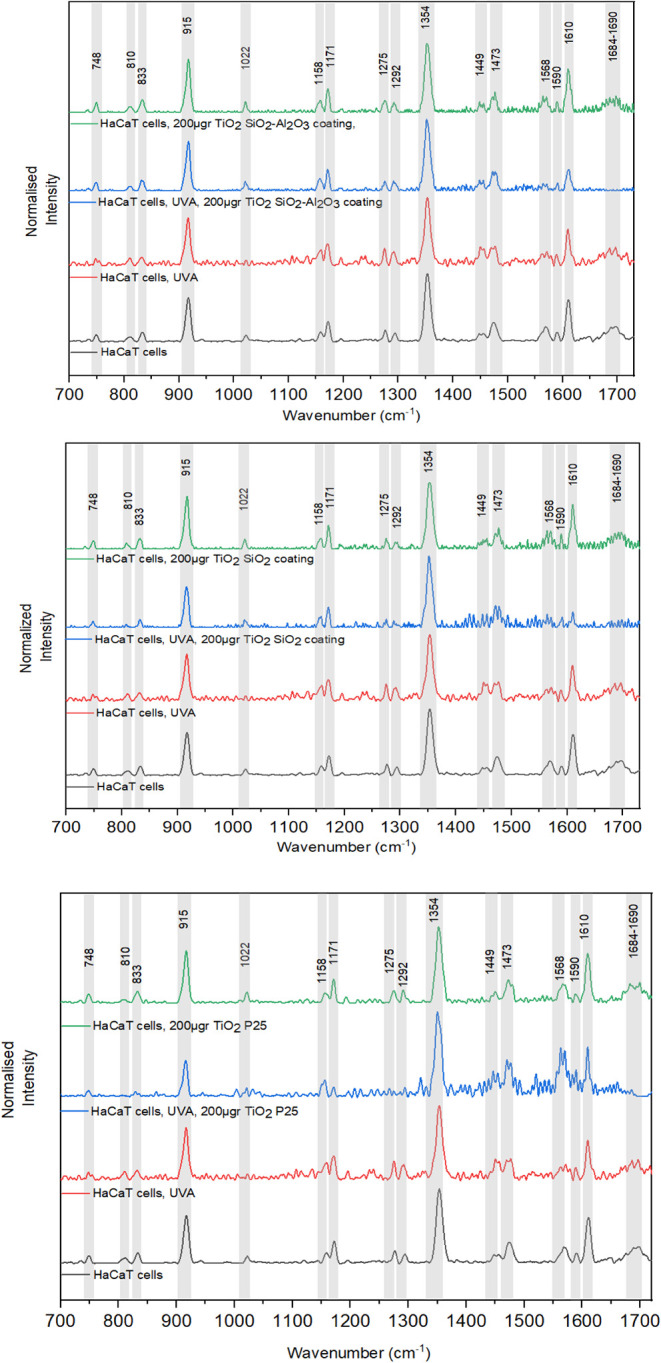
Raman spectra of HaCaT cells treated with uncoated and coated anatase/rutile mixtures of nano-TiO_2_ and/or UVA.

Table 2 summarizes the characteristic Raman shifts of cellular components based on previously published data. Several spectral features remain unchanged across all conditions, including strong peaks at 915 cm^–1^ and 1354 cm^–1^, corresponding to proline/hydroxyproline and adenine/guanine (DNA), as well as tryptophan (proteins), respectively. Additional stable peaks appear at 810, 833, 1158, 1171, 1275, 1292, 1473, and 1590 cm^–1^, which are typically associated with DNA, proteins, and lipids. Notable differences were also observed. The weak peak at 1022 cm^–1^, which is absent in the UVA only treated cells, may indicate UVA-induced photo-oxidative damage, likely due to ROS production that has been linked to apoptosis and DNA/protein degradation.^15^ The absence of the peak at 1022 cm^–1^ in UVA-exposed samples suggests photodegradation of vibrational modes associated with phenylalanine or methoxy-containing groups,^48^ likely from protein or amino acid structures.^49^ Notably, this peak remains detectable in samples treated with anatase-based TiO_2_ nanoparticles, implying a potential protective effect. Classical molecular dynamics (MD) simulations support this observation by demonstrating that phenylalanine (Phe) adsorbs onto the surface of neutral TiO_2_ nanoparticles. Phe forms stable interactions through its aromatic ring, with potential of mean force (PMF) calculations revealing a favorable adsorption energy (∼10 kJ/mol).^50^ Additionally, simulations indicate that neutralizing TiO_2_ surface charges disrupts the interfacial water layer, potentially enhancing hydrophobic interactions and promoting aromatic amino acid adsorption.^50^ Although fully neutral TiO_2_ is not physiologically representative, these findings provide mechanistic insight into how TiO_2_ nanoparticles may stabilize specific biomolecules, thereby mitigating UVA-induced photodegradation.

**Table 2:** Assignment of Spectra Bands in HaCaT Cells

Raman shift (cm^–1^)	Assignment
748	Tryptophan,^41^ T, DNA bases^42^
810	Phosphodiester (Z-marker) DNA^43^
833	Proline, hydroxoproline, tyrosine, v2 PO_2_ stretch of nucleic acids,^41^ amide III^42^
915	Proline, hydroxyproline^41,43^
1022	O–CH_3_ stretching of methoxy groups,^43^ phenylalanine^43^
1158	νC–C, lipid chains^43,44^
1171	Cytosine, guanine |DNA|,^43^ C–H in plane bend tyrosine |protein| ^43,45^
1275	Amide III of collagen, amide III (collagen assignment), amide III, ν(CN), δ(NH) amide III, α-helix, collagen (protein assignment)^45^
1292	δCH_2_ twisting (lipid), lipid^44,45^
1354	Adenine, guanine |DNA| tryptophan |protein|^41^
1449	CH_2_ bending mode of proteins. CH_2_ (overlapping) asymmetric CH_3_ bending, and CH_2_ scissoring (associated with elastin, collagen, and phospholipids)^46^
1473	CH_2_CH_3_ deformation^45^
1568	Ring breathing modes in the DNA bases, G, A (ring breathing modes of the DNA/RNA bases),^45^ tryptophan^44^
1590	Hydroxyproline,^41^ phenylalanine^45^
1610	Tyrosine, C=C (protein),^41,45^ phenylalanine,^42^ tryptophan^45^
1684–1690	ν(C=O) amide I,^47^ α helix of keratin, ν(C=C) lipid^44^

A pronounced reduction or complete disappearance of the amide I peak (1684–1690 cm^–1^), associated with proteins, was observed in cells exposed to both anatase-based TiO_2_ nanoparticles and UVA radiation, respectively. Although NPs have been detected in various intracellular compartments—including the endoplasmic reticulum,^51^ cytoplasm, and nucleus^10^—the mechanisms by which they penetrate cellular barriers remain unclear. One plausible explanation involves interactions between TiO_2_-induced reactive oxygen species (ROS) and cellular proteins and lipids.

ROS can induce lipid peroxidation and protein degradation,^52^ as evidenced by the loss of some peaks that are associated with lipid and protein when both UVA and anatase-based TiO_2_ NPs are present. Since the cell membrane is composed primarily of lipids and proteins,^53^ ROS-mediated damage to these structures may facilitate nanoparticle internalization.^10^ The so-called “Trojan-horse” mechanism, in which nanoparticles are internalized by cells and subsequently release high concentrations of toxic ions,^54^ may also apply to TiO_2_ nanoparticles. This intracellular release is a potential contributor to their observed cytotoxic effects.

The reduced intensity of the peaks at 1568 cm^–1^ and 1610 cm^–1^ of the cell cultures treated with the coated anatase-based nano-TiO_2_ and UVA, corresponding to tryptophan, is consistent with literature reports indicating that oxide nanoparticles can disrupt tryptophan’s amide bonds.^55^ Tryptophan is an essential amino acid involved in protein synthesis, cell survival, and key metabolic functions.^56,57^ Additionally, the 1568 cm^–1^ and 810 cm^–1^ peaks-associated with DNA base ring breathing modes-also exhibited diminished intensity in cells treated with both nanoparticles and UVA, suggesting potential structural disruption of DNA under these conditions.

However, the peak at 1610 cm^–1^ remained unaffected in cell cultures treated with uncoated anatase-based nanoparticles. We attribute this observation, at least in part, to the difficulty in obtaining enough viable cells for Raman measurement in these samples. As demonstrated by the XTT cytotoxicity results, cultures treated with uncoated anatase-based particles exhibited markedly reduced viability compared to those treated with coated anatase-based particles.

Our proposed mechanism suggests that the interaction between TiO_2_ nanoparticles and cellular components is time dependent. In the case of coated anatase-based nanoparticles, this interaction proceeds more slowly, allowing a greater proportion of cells to remain viable for Raman analysis and thus enabling the detection of amino acid and protein-associated peaks. In contrast, uncoated anatase-based nanoparticles induce more rapid and extensive damage, leaving fewer intact cells available for measurement, which may explain the absence of detectable alterations at 1610 cm^–1^ in this case.

## 4 Conclusions

In this study, we investigated how surface coatings influence the photocatalytic activity and cytotoxicity of anatase-based TiO_2_ nanoparticles, which are widely used as UV filters in sunscreens. Photocatalytic activity was assessed via methylene blue degradation, driven by reactive oxygen species (ROS) generated upon UV irradiation when anatase-based TiO_2_ NPs are also present. The results showed that uncoated anatase-based TiO_2_ nanoparticles, namely, Aeroxide P25, displayed higher photocatalytic activity compared to coated forms. In contrast, SiO_2_ and Al_2_O_3_–SiO_2_ coatings markedly reduced the photocatalytic efficiency, indicating that surface modifications can significantly alter ROS generation at the nanoparticle surface. These differences highlight the importance of nanoparticle surface chemistry in practical applications, such as sunscreen formulations.

Cytotoxicity studies revealed that UVA irradiation alone did not significantly affect the HaCaT cell viability. However, in the presence of anatase-based TiO_2_ nanoparticles, UVA exposure induced pronounced cytotoxicity in a dose- and time-dependent manner. Among the tested coatings, Al_2_O_3_–SiO_2_ provided the greatest protective effect, substantially reducing but not eliminating cytotoxicity, consistent with the photocatalysis results. ROS measurements further confirmed that nanoparticle-treated cells experienced elevated oxidative stress compared with untreated controls. Finally, Raman spectroscopy demonstrated the loss of characteristic lipid- and protein-associated peaks in cells exposed to both UVA and anatase-based TiO_2_ nanoparticles, suggesting membrane disruption that could compromise cellular integrity and expose DNA to damage. Overall, these findings underscore the dual role of nanoparticle coatings in reducing both the photocatalytic activity and cytotoxic effects. Such considerations are critical for the safe design and selection of TiO_2_ nanoparticles in sunscreen formulations by the manufacturers.

### 4.1 Limitations and Further Work

The present study did not involve the testing of as-formulated sunscreen products (or TiO_2_ extracted from them), the use of 2D HaCaT cells instead of skin with barrier/immune components, and the application of UVA-only spectra in simple media. A real-world assessment should entail the examination of as-formulated particles at SPF-relevant film loads under solar-simulated UVA and UVB on reconstructed/ex vivo human skin, with the objective of quantifying Ti penetration and inflammatory markers.

## Supplementary Material



## Notes

XRD patterns of the investigated anatase-dominant TiO_2_ nanoparticle variants, including uncoated, SiO_2_-coated, and Al_2_O_3_–SiO_2_-coated materials (Figure S1) (PDF)
