# Supplementary material for: Impact of Surface Coatings on the Photocatalytic Activity and Cytotoxicity of Titanium Dioxide Nanoparticles in Human Keratinocytes: Implications for Sunscreen Safety
Source: ACS Omega. 2026 Jun 5;11(24):35180–90. doi: 10.1021/acsomega.5c13018 (PMC13294874; doi:10.1021/acsomega.5c13018)
Supplement: Supplementary file 1 [file ao5c13018_si_001.pdf]

Supporting Information for

Impact of Surface Coatings on the Photocatalytic Activity and Cytotoxicity of Titanium Dioxide Nanoparticles in Human Keratinocytes: Implications for Sunscreen Safety

Maria Eleni Katsanou[1*], Philipp-Kjell Ficht[2], Lienhard Wegewitz[1], Jörg Adams[3], Lars Böckmann[2], Steffen Emmert[2], Wolfgang Maus-Friedrichs[1]

[1] Clausthaler Center for Materials Technology, Clausthal University of Technology, Germany; [2] Clinic and Policlinic for Dermatology, Venereology and Allergology, University Medical Center Rostock, Germany; [3] Institute of Physical Chemistry Clausthal University of Technology, Germany

**Corresponding author at Clausthaler Center for Materials Technology, Clausthal University of Technology, Germany*

E-mail address: maria.eleni.katsanou@tu-clausthal.de

XRD Characterization of the samples

These data were added to provide structural characterization of the studied TiO_2 samples and to better document their crystal phase composition. X-ray diffraction (XRD) analysis was performed using an Empyrean diffractometer (Malvern Panalytical) equipped with a Cu Kα radiation source. The crystal phase composition of the samples was determined from the diffraction patterns, and quantitative phase analysis of the anatase/rutile mixtures was carried out using the Spurr and Myers equation[1].

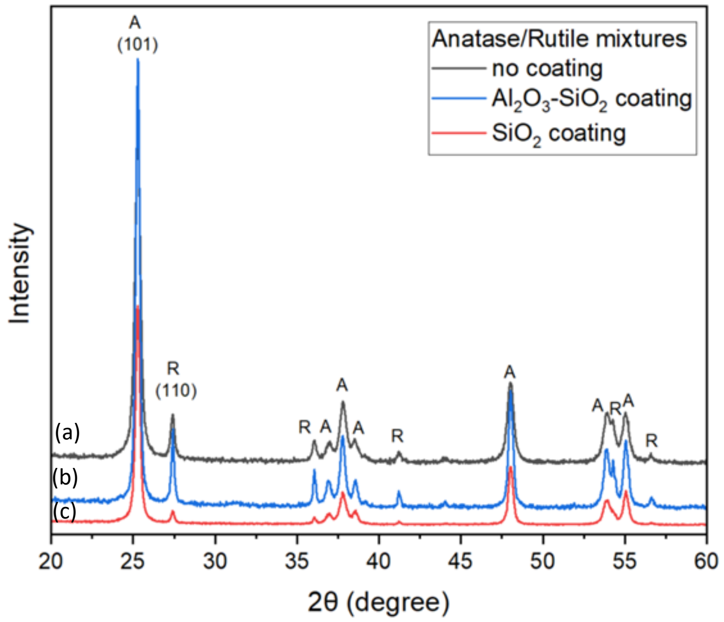

Figure S1: XRD analysis of anatase/rutile mixtures (a) without coating (black), with (b) Al_2O_3-SiO_2 coating (blue) and (c) SiO_2 coating (red).

More specifically the ratio calculated using intensity of the main peaks of anatase and rutile (101), (110) as highlighted in Figure S1. The ratio for Evonik Aeroxide TiO_2 P25 and for Al_2O_3-SiO_2 coated TiO_2 NPs are 80% Anatase: 20% Rutile, but for SiO_2 coated TiO_2 nanoparticles ratio is 90% Anatase: 10% Rutile.

References

(1) Spurr, R. A.; Myers, Howard. Quantitative Analysis of Anatase-Rutile Mixtures with an X-Ray Diffractometer. *Anal. Chem.* **1957**, *29* (5), 760–762. https://doi.org/10.1021/ac60125a006.
